# Hybrid Calcium Hydroxylapatite–Polynucleotide Skin Booster A Retrospective Cohort Study

**DOI:** 10.1111/jocd.70897

**Published:** 2026-06-08

**Authors:** Andrea Lazzarotto, Stefania Guida, Jonathan Kadouch, Nabil Fakih‐Gomez, Nicola Zerbinati, Luigi Colombo, Massimo Vitale, Mariana César Corrêa, Kyu‐Ho Yi

**Affiliations:** ^1^ Department of Maxillofacial and Plastic Surgery University Hospital ASST‐Lariana Como Italy; ^2^ Private Practice Como Italy; ^3^ Private Practice Milan Italy; ^4^ Private Practice Udine Italy; ^5^ School of Medicine Vita‐Salute San Raffaele University Milan Italy; ^6^ Dermatology Clinic IRCCS San Raffaele Scientific Institute Milan Italy; ^7^ Practice for Aesthetic Dermatology ReSculpt Clinic Amsterdam the Netherlands; ^8^ Department of Facial Plastic and Cranio‐Maxillo‐Facial Surgery Fakih Hospital Khaizaran Lebanon; ^9^ Department of Medicine and Surgery University of Insubria Varese Italy; ^10^ Maxillofacial and Plastic Surgery Department University Hospital “ASST‐Lariana” of Como Como Italy; ^11^ Private Practice Bologna Italy; ^12^ Clinica Dermatologica Dra, Mariana Corrêa São Paulo Brazil; ^13^ You and I Clinic Seoul Republic of Korea

**Keywords:** calcium hydroxylapatite, collagen biostimulation, facial rejuvenation, GAIS, polynucleotide, skin booster, skin quality

## Abstract

**Background:**

Skin quality is increasingly recognized as a multidimensional construct that includes tone evenness, surface evenness, firmness, and radiance. Injectable ‘skin boosters’ have expanded treatment options for diffuse skin‐quality concerns, and combinations of biostimulatory and regenerative agents are increasingly used in practice.

**Objective:**

To evaluate the 6‐month effectiveness and safety of a single‐session hybrid calcium hydroxylapatite–polynucleotide (CaHA–PN) skin booster protocol using diluted CaHA and PN in a consecutive real‐world cohort.

**Methods:**

A single‐center, retrospective observational cohort analysis included 36 consecutive patients treated with a fixed‐dose hybrid protocol. Diluted calcium hydroxylapatite (1.5 mL CaHA (FACETEM, CGBio Inc., Korea), diluted 1:2 with preservative‐free normal saline) was injected in the subdermal plane with a 25‐gauge blunt cannula, followed by intradermal polynucleotide (DOT‐PN, Rejuran, Pharmaresearch; 4.0 mL) administered via micro‐aliquots using a 32‐gauge needle. Standardized photographs were obtained at baseline and 6 months. Two blinded dermatologists graded facial skin quality using a validated 5‐point photonumeric scale (0–4). Secondary outcomes included cheek wrinkle severity (Modified Fitzpatrick Wrinkle Scale framework), clinician‐ and patient‐rated Global Aesthetic Improvement Scale (GAIS), patient‐reported radiance (Likert 1–5), satisfaction, and adverse events.

**Results:**

The mean age was 45.8 ± 12.3 years (range 17–68) and 32/36 (88.9%) were female. At 6 months, the mean skin quality score improved from 2.7 ± 0.8 to 1.5 ± 0.6 (44.4% improvement; *p* < 0.001), and mean cheek wrinkle severity improved from 2.4 ± 0.9 to 1.4 ± 0.5 (41.7% improvement; *p* < 0.001). GAIS indicated improvement (scores 1–3) in 83.3% and 80.6% of clinician assessments and in 91.7% of patient self‐assessments (all *p* < 0.001). High radiance (Likert ≥ 4) was reported by 86.1% of patients, and 77.8% reported being satisfied or very satisfied at 6 months; 94.4% would recommend the treatment. Transient injection‐site erythema (83.3%), edema (77.8%), and ecchymosis (41.7%) were the most frequent adverse events. Two patients (5.6%) developed delayed nodules that resolved with massage by 8 weeks. No infections, vascular compromise, or serious adverse events were observed.

**Conclusion:**

In this 36‐patient cohort, a single‐session hybrid CaHA–PN skin booster protocol was associated with significant improvements in facial skin quality and cheek wrinkle severity at 6 months, with high patient‐reported improvement and a favorable safety profile. Prospective controlled studies are needed to define comparative efficacy and to optimize dosing schedules.

## 1 Introduction

Interest in ‘skin quality’ has accelerated over the past decade as aesthetic practice has shifted from treating isolated wrinkles toward achieving balanced, global facial rejuvenation. Consensus statements describe skin quality as a multidimensional construct that encompasses visible features such as surface texture and pore appearance, tone evenness and radiance, and mechanical properties such as firmness and elasticity [1, 2]. From a patient perspective, improvements in radiance and surface smoothness may be as important as the reduction of discrete rhytides, particularly among individuals who wish to avoid overt volumization or surgical interventions.

Skin quality declines through both intrinsic aging and extrinsic injury. Chronologic aging is associated with progressive alterations in extracellular matrix (ECM) homeostasis, including reductions in collagen content and changes in elastin organization, while environmental exposures (most notably ultraviolet radiation and pollution) contribute to oxidative stress, inflammation, and dysregulated remodeling. These processes can manifest clinically as rougher texture, larger visible pores, reduced glow, and mild laxity. Because multiple epidermal and dermal pathways contribute to these phenotypes, a single treatment modality rarely addresses the full spectrum of skin‐quality concerns. Consequently, minimally invasive ‘skin booster’ strategies have expanded, including injectables designed to improve dermal structure and hydration with limited downtime.

Among biostimulatory injectables, calcium hydroxylapatite (CaHA) has an extensive safety record and broad clinical experience [3, 4]. While CaHA has traditionally been used for volumization and contouring, contemporary practice increasingly applies CaHA in dilute or hyperdilute formulations to target dermal biostimulation rather than focal volumetric correction. In this context, CaHA can be distributed across wider facial regions in superficial planes, supporting fibroblast activity and collagen remodeling. Expert consensus recommendations provide guidance on dilution ratios, injection depth, and mapping for CaHA when used as a biostimulatory agent for the face and body [5]. Clinical studies using diluted CaHA have reported improvements in skin mechanical properties, and ultrasonographic work has demonstrated increased dermal thickness after treatment in selected anatomical regions [6, 7].

Polynucleotides (PN) have emerged as a regenerative class of injectable skin boosters. PN products generally contain highly purified DNA fragments and are proposed to support tissue repair by scaffold‐like properties. Recent reviews summarize growing clinical use of PN in facial rejuvenation, noting potential improvements in wrinkles, texture, elasticity, and overall appearance [8]. A systematic review found that PN injections show promising outcomes in reducing wrinkles and improving skin texture by its scaffold effect, although study heterogeneity and variable methodological quality limit firm conclusions [9]. Prospective clinical investigations include randomized and controlled designs, such as a phase III matched‐pairs trial comparing PN filler with hyaluronic acid filler for correction of crow's feet, with favorable durability and safety outcomes for PN [10]. Additional prospective work suggests PN can be combined with other injectables (e.g., cross‐linked hyaluronic acid) to enhance clinical effect while maintaining tolerability [11].

Given the complementary therapeutic rationale—CaHA as a durable biostimulatory substance and PN as a scaffold—hybrid CaHA–PN protocols are increasingly used to address multiple skin‐quality domains in a single visit. Despite widespread adoption, published data on fixed‐dose CaHA–PN ‘hybrid skin booster’ protocols, particularly with mid‐term follow‐up, remain limited. Related ‘hybrid’ concepts have been described for CaHA combined with hyaluronic acid gels, where a premixed CaHA/CMC–hyaluronic acid hybrid filler improved both skin quality and wrinkle severity in a small cohort [12]. However, CaHA–PN protocols differ substantially in biological target and injection strategy, highlighting the need for data specific to CaHA and PN combinations.

The objective of this study was to evaluate the 6‐month effectiveness and safety of a single‐session, fixed‐dose hybrid CaHA–PN skin booster regimen (1.5 mL diluted CaHA + 4.0 mL intradermal PN) in 36 consecutive patients using validated and clinically meaningful outcome measures.

## 2 Materials and Methods

### 2.1 Study Design and Reporting Framework

This was a single‐center, retrospective observational cohort analysis performed in a routine clinical practice setting. The study report was prepared with reference to the Strengthening the Reporting of Observational Studies in Epidemiology (STROBE) guidance for observational research [13].

### 2.2 Ethics, Consent, and Confidentiality

All patients provided written informed consent for treatment. For the purposes of this analysis, all data were de‐identified prior to extraction and analysis. All patients provided written consent for clinical photography and for publication of de‐identified images.

### 2.3 Participants

Consecutive patients who underwent the hybrid CaHA–PN skin booster procedure and returned for standardized 6‐month follow‐up photography were eligible for inclusion. A total of 36 consecutive patients met eligibility criteria.

Inclusion criteria were as follows:

(1) age 17–68 years.

(2) desire for improvement in global facial skin quality (including one or more of texture roughness, reduced radiance, visible pores, or mild laxity).

(3) availability of standardized baseline and 6‐month follow‐up photographs.

(4) no additional facial procedures during the 6‐month follow‐up interval (including energy‐based devices, injectables, or facial surgery).

Exclusion criteria were as follows:

(1) active skin infection or inflammatory dermatosis in the planned treatment area.

(2) pregnancy or lactation.

(3) known hypersensitivity to CaHA or PN components.

(4) a history of autoimmune disease or current immunosuppressive therapy.

(5) facial surgery or energy‐based treatments within the 6 months prior to treatment.

### 2.4 Injectable Products

Two injectable products were used in a fixed‐dose regimen.

**FIGURE 1:**
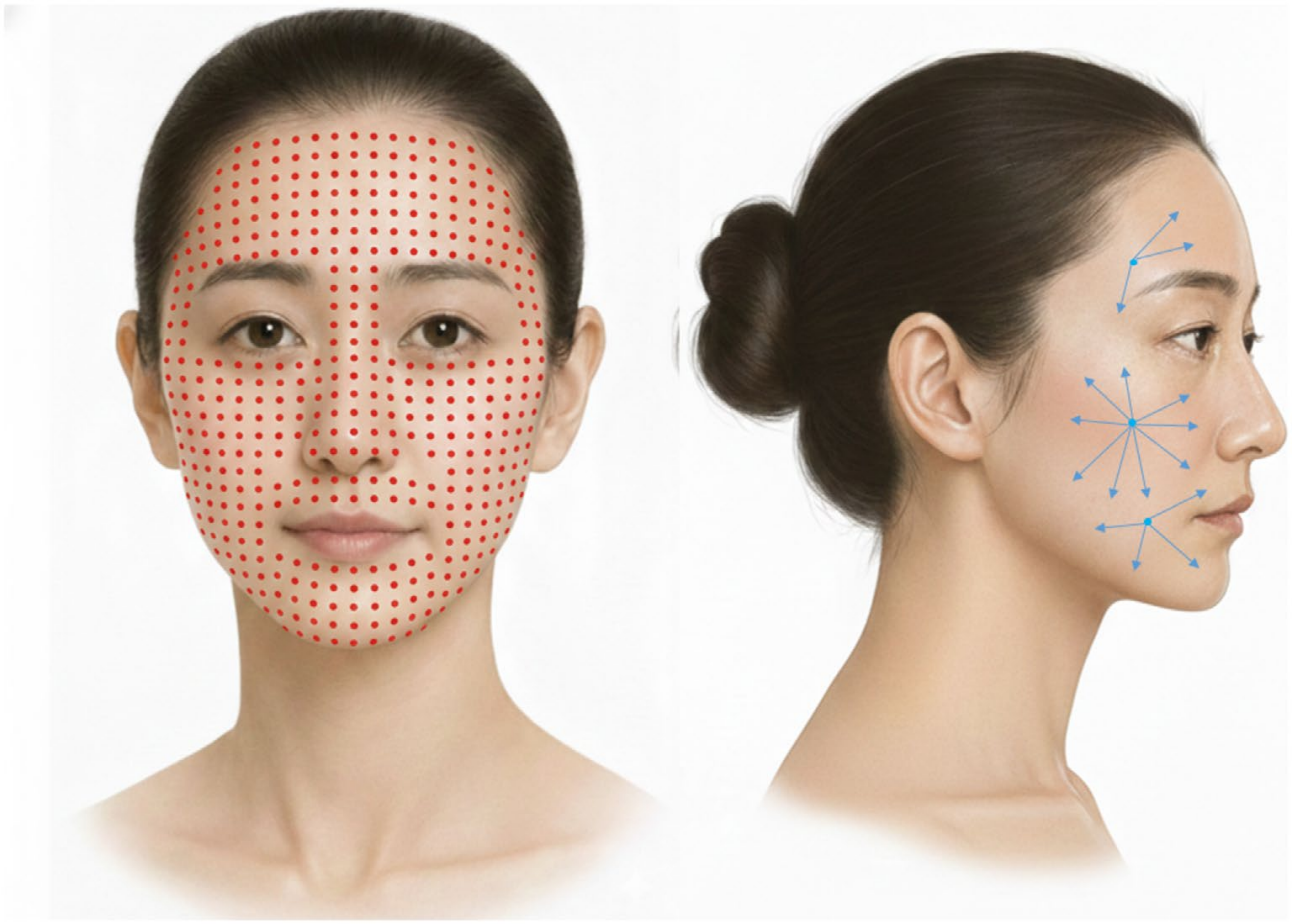
The intradermal injection of PN (red dots) and subdermal injection of Hyperdiluted CaHA (Blue arrows) were delivered for skin quality improvement.

Calcium hydroxylapatite (CaHA): A 1.5‐mL syringe of calcium hydroxylapatite (FACETEM, CGBio Inc., Korea) was diluted at a 1:2 ratio with preservative‐free normal saline immediately prior to treatment. This dilution strategy was selected to facilitate broad subdermal distribution for skin‐quality biostimulation rather than focal volumetric augmentation, consistent with contemporary biostimulatory use concepts for CaHA [5] (Figure 1).

Polynucleotide (PN): A total of 4.0 mL of DOT‐PN (DNA Optimizing Technology Polynucleotide; Rejuran, PharmaResearch Products, Seoul, Republic of Korea) was used per session. PN products have been used as injectable biorevitalizers in aesthetic medicine and have been reported to improve texture and fine lines in multiple clinical contexts [8, 9, 10, 11] (Figure 1).

### 2.5 Treatment Protocol

All treatments were performed by a single experienced injector to reduce variability related to technique. Topical anesthetic containing lidocaine 5% was applied to the treatment area and removed prior to skin preparation. The face was cleansed and disinfected using standard antiseptic technique.

Step 1 (subdermal CaHA): Diluted CaHA (FACETEM, CGBio Inc., Korea) was injected in the subdermal plane using a 25‐gauge blunt cannula. Entry points were selected to enable even product fanning across mid‐ and lower‐face target regions. The injectate was placed using retrograde linear threads with gentle, low‐pressure delivery to minimize the risk of bolus deposition. The total CaHA volume was 1.5 mL per session, distributed bilaterally across the malar region, perioral region, and jawline.

Step 2 (intradermal PN): DOT‐PN (Rejuran, Korea) was delivered intradermally using a 32‐gauge needle employing a micro‐aliquot technique. Small aliquots were placed at short intervals to achieve even dermal coverage, with attention to avoiding intravascular injection and minimizing product pooling. The total PN volume was 4.0 mL per session, distributed bilaterally across the same target regions.

Following injection, gentle manual molding was performed as needed to optimize distribution and to reduce focal irregularities. Patients were provided with standard post‐procedure instructions including avoidance of vigorous massage, excessive heat exposure, and strenuous exercise for 24 h, and were advised to report any persistent pain, progressive erythema, or delayed nodularity.

### 2.6 Photography Standardization

Standardized photographs were obtained at baseline (pre‐treatment) and at 6 months using a fixed‐distance facial imaging system with head positioning to minimize variation in angle, distance, and lighting. Frontal and oblique views were acquired in a consistent sequence. All photographic comparisons and figure images in this manuscript are representative standardized images captured under the same acquisition conditions at both time points.

### 2.7 Outcome Assessment

Outcomes were assessed at baseline and at 6 months.

The primary efficacy endpoint was the change in facial skin quality from baseline to 6 months using a validated 5‐point photonumeric scale (0 = best skin quality; 4 = poorest skin quality) assessed from standardized photographs [14].

Two board‐certified dermatologists who were not involved in treatment independently evaluated all images in a blinded manner. To reduce assessment bias, baseline and follow‐up images were presented in randomized order and assessed under standardized viewing conditions.

The final score for each patient at each time point was calculated as the mean of the two independent ratings. Inter‐rater reliability was assessed using Cohen's kappa (κ), which demonstrated substantial agreement (κ = 0.71). Although kappa was used as a measure of categorical agreement, the use of a validated photonumeric scale and standardized assessment conditions was considered sufficient for exploratory evaluation in this real‐world study.

(1) Wrinkle severity: Cheek wrinkle severity was assessed using an ordinal wrinkle severity scale adapted from the Modified Fitzpatrick Wrinkle Scale (MFWS), which is originally validated for nasolabial folds [15].

In the absence of a validated cheek‐specific wrinkle scale, the MFWS framework was applied to the cheek region using analogous depth‐based descriptors to ensure consistent grading across time points. This adapted use was intended to provide a standardized reference for within‐subject comparison rather than absolute wrinkle quantification.

(2) Global Aesthetic Improvement Scale (GAIS): Global improvement was assessed at 6 months by two clinicians and by patients using the 5‐point GAIS (1 = Very Much Improved, 2 = Much Improved, 3 = Improved, 4 = No Change, 5 = Worse) [16]. ‘Improved’ was defined a priori as GAIS scores 1–3.

(3) Patient‐reported radiance: Patients rated perceived facial radiance on a 5‐point Likert scale (1 = very low radiance to 5 = very high radiance).

(4) Patient satisfaction: Patients recorded satisfaction using a 5‐point satisfaction response (very satisfied, satisfied, neutral, dissatisfied, very dissatisfied) at 6 months.

(5) Safety: Safety outcomes included immediate injection‐site reactions (erythema, edema, ecchymosis), procedure‐related pain measured by a 0–10 visual analog scale (VAS), and delayed adverse events (including nodules, infection, dyspigmentation, and vascular compromise).

### 2.8 Statistical Analysis

Descriptive statistics are reported as means ± standard deviation (SD) for continuous variables and counts/percentages for categorical variables. Ordinal outcomes (skin‐quality score, wrinkle severity score, and GAIS) were analyzed using the Wilcoxon signed‐rank test. Continuous outcomes (e.g., pain VAS) were analyzed using paired *t*‐tests when appropriate. Statistical significance was set at *p* < 0.05 (two‐sided).

Given the ordinal nature of the rating scales, non‐parametric statistical methods were primarily applied to evaluate treatment effects.

Inter‐rater agreement between the two blinded dermatologist raters was evaluated using Cohen's kappa (κ) statistic, a chance‐corrected measure of categorical agreement [17].

## 3 Results

The cohort included 36 patients with a mean age of 45.8 ± 12.3 years (range 17–68 years). Female patients comprised 32/36 (88.9%). Fitzpatrick skin phototypes were II in 14 patients (38.9%), III in 16 (44.4%), and IV in 6 (16.7%). All patients presented with a primary desire to improve global facial texture and radiance. Visible pores, subtle surface roughness, and mild laxity were commonly noted at baseline during routine clinical evaluation. Twenty of 36 patients (55.6%) reported a history of prior aesthetic procedures, reflecting a typical real‐world population seeking adjunctive skin‐quality improvement. The mean baseline photonumeric skin‐quality score was 2.7 ± 0.8. Baseline demographic and clinical characteristics are summarized in Table 1.

**TABLE 1:** Baseline characteristics (*n* = 36).

Characteristic	Value
Age, mean ± SD (range)	45.8 ± 12.3 (17–68) years
Sex, *n* (%) female	32 (88.9%)
Fitzpatrick skin type, *n* (%)	II: 14 (38.9%); III: 16 (44.4%); IV: 6 (16.7%)
Baseline skin quality score (0–4)	2.7 ± 0.8
Primary indication: texture/radiance	36 (100%)
Prior aesthetic procedures	20 (55.6%)

**FIGURE 2:**
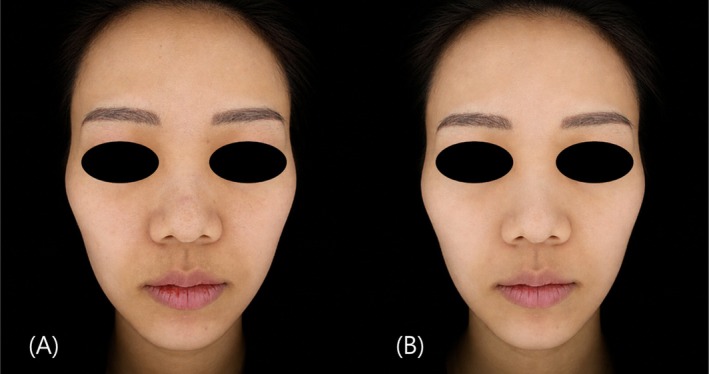
Representative standardized frontal photographs at baseline (left) and 6 months (right) after a single hybrid CaHA–PN skin booster session.

**FIGURE 3:**
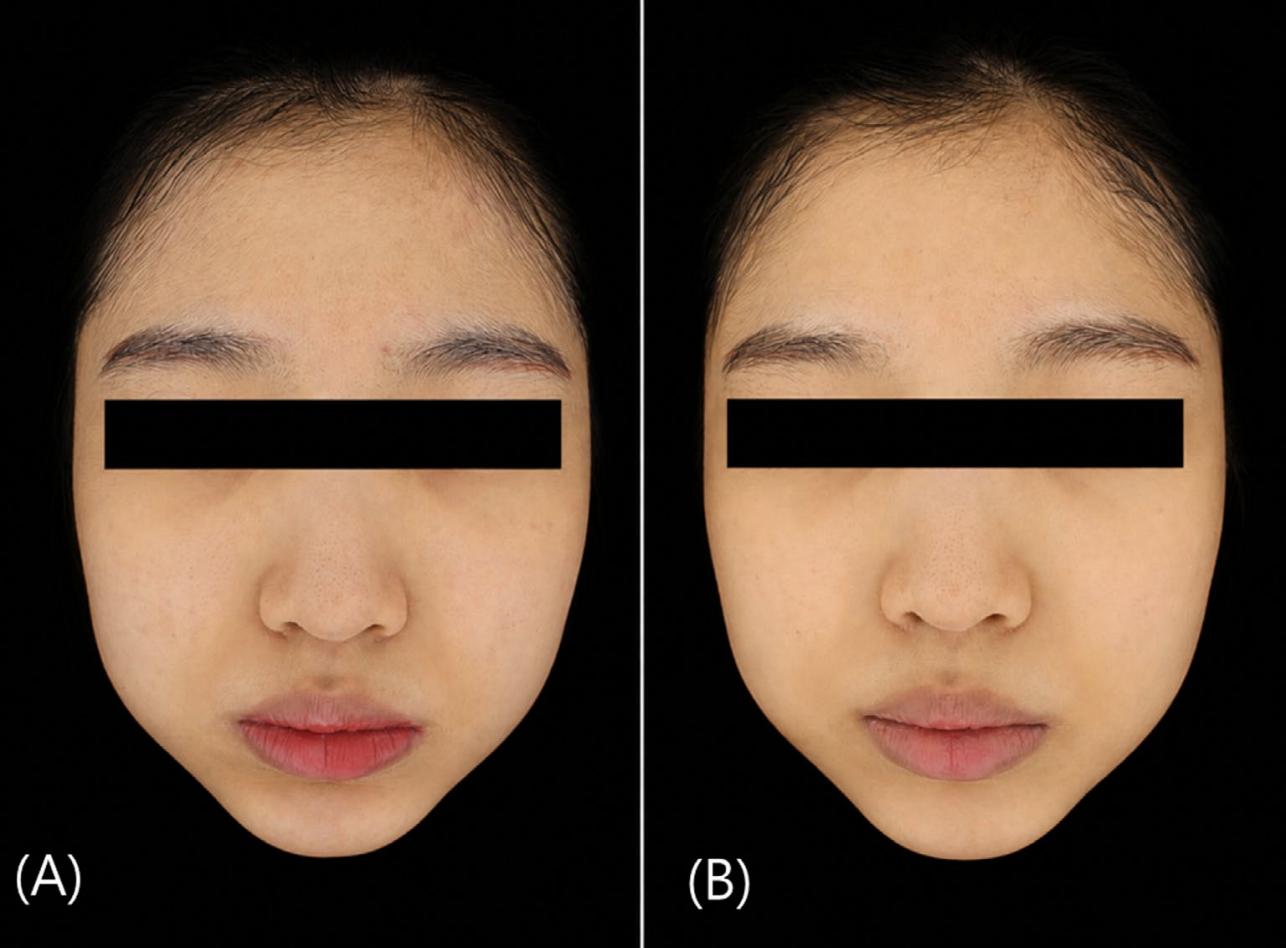
Representative standardized frontal photographs at baseline (left) and 6 months (right) after treatment.

**FIGURE 4:**
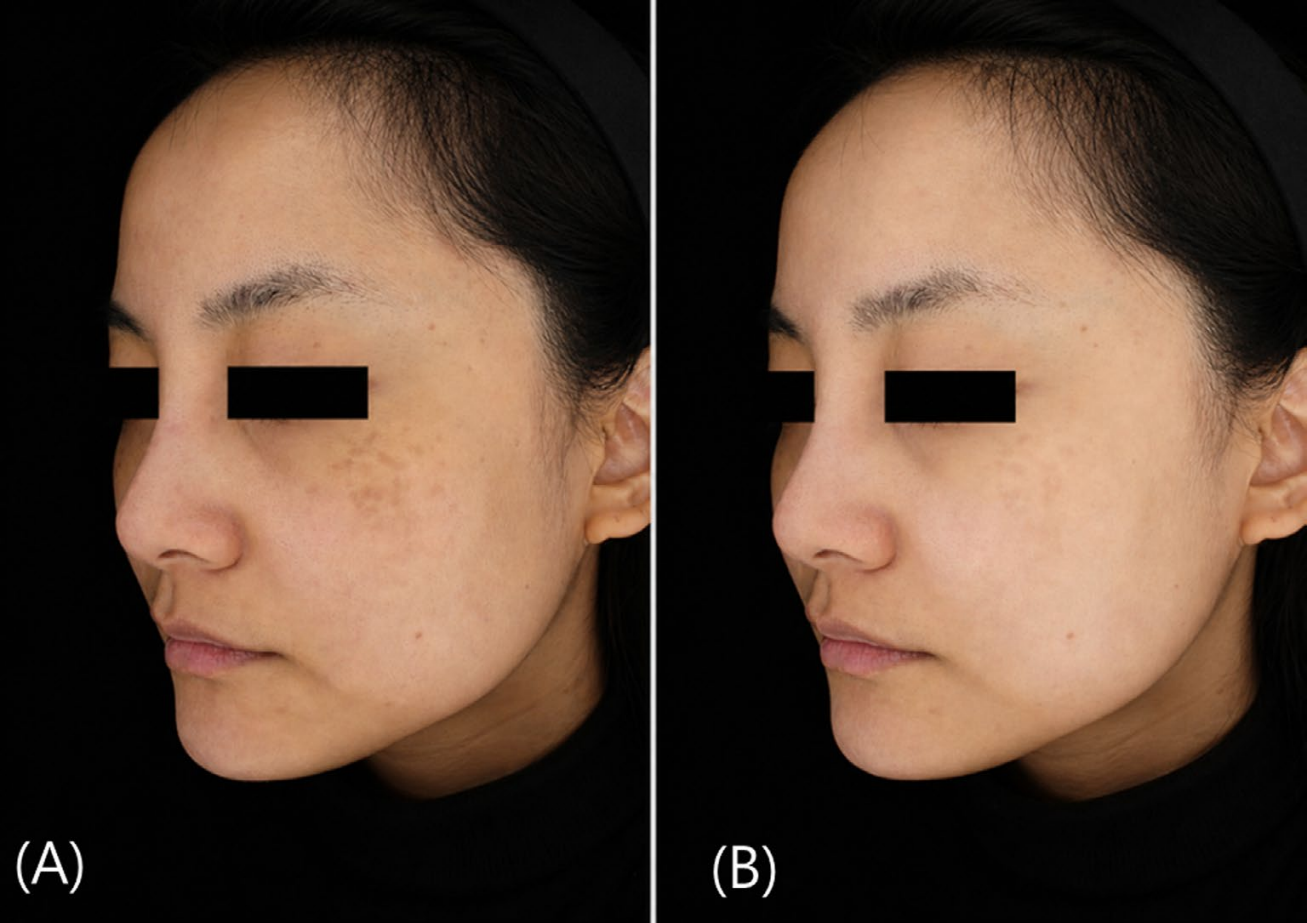
Representative standardized oblique photographs at baseline (left) and 6 months (right) after treatment.

At 6 months following a single hybrid CaHA–PN session, the mean photonumeric skin‐quality score improved from 2.7 ± 0.8 at baseline to 1.5 ± 0.6, corresponding to a 44.4% improvement (*p* < 0.001; Table 2). Both blinded dermatologist raters identified improvement across the cohort, and inter‐rater agreement for categorical grading was substantial (κ = 0.71). Representative standardized clinical photographs illustrating changes in overall facial appearance are shown in Figures 2, 3, 4.

**TABLE 2:** Efficacy outcomes at 6 months (baseline vs follow‐up).

Outcome measure	Baseline (T0)	Follow‐up (T1)	Change/improvement	*p*
Skin quality score (0–4)	2.7 ± 0.8	1.5 ± 0.6	44.4%	< 0.001
Wrinkle severity – cheek (0–5)	2.4 ± 0.9	1.4 ± 0.5	41.7%	< 0.001
GAIS – clinician 1, *n* (%) improved	—	30 (83.3%)	—	< 0.001
GAIS – clinician 2, *n* (%) improved	—	29 (80.6%)	—	< 0.001
GAIS – patient, *n* (%) improved	—	33 (91.7%)	—	< 0.001
Patient‐reported radiance (Likert ≥ 4, 1–5)	—	31 (86.1%)	—	< 0.001

*Note:* Improved was defined as GAIS score 1–3 (Very Much Improved, Much Improved, Improved).

### 3.1 Cheek Wrinkle Severity

Cheek wrinkle severity improved significantly at 6 months. The mean wrinkle severity score decreased from 2.4 ± 0.9 at baseline to 1.4 ± 0.5 at follow‐up, representing a 41.7% improvement (*p* < 0.001; Table 2).

### 3.2 Global Aesthetic Improvement Scale (GAIS)

Clinician‐rated GAIS indicated improvement (scores 1–3) in 30/36 patients (83.3%) for clinician 1 and 29/36 patients (80.6%) for clinician 2 (both *p* < 0.001). Patient‐rated GAIS indicated improvement in 33/36 patients (91.7%; *p* < 0.001; Table 2). No patient was rated as “Worse” (GAIS 5) by any evaluator.

A detailed GAIS category distribution is presented in Table 3. Among clinician 1 ratings, 9/36 (25.0%) were “Very Much Improved” and 14/36 (38.9%) were “Much Improved.” Among clinician 2 ratings, 8/36 (22.2%) were “Very Much Improved” and 13/36 (36.1%) were “Much Improved.” Patients most frequently selected higher improvement categories: 15/36 (41.7%) self‐rated as “Very Much Improved” and 13/36 (36.1%) self‐rated as “Much Improved.”

**TABLE 3:** GAIS category distribution at 6 months (*n* = 36).

GAIS category	Clinician 1	Clinician 2	Patient
Very much improved (1)	9 (25.0%)	8 (22.2%)	15 (41.7%)
Much improved (2)	14 (38.9%)	13 (36.1%)	13 (36.1%)
Improved (3)	7 (19.4%)	8 (22.2%)	5 (13.9%)
No change (4)	6 (16.7%)	7 (19.4%)	3 (8.3%)
Worse (5)	0 (0%)	0 (0%)	0 (0%)
Total improved (1–3)	30 (83.3%)	29 (80.6%)	33 (91.7%)

*Note:* Inter‐rater agreement between clinicians: κ = 0.71 (substantial agreement).

### 3.3 Patient‐Reported Radiance and Satisfaction

At 6 months, 31/36 patients (86.1%) reported high radiance (Likert ≥ 4). Overall satisfaction was favorable: 28/36 (77.8%) reported being “satisfied” or “very satisfied,” while 34/36 (94.4%) indicated they would recommend the treatment to others.

### 3.4 Safety Outcomes

The safety profile was favorable and consistent with expected post‐injection reactions for combined cannula‐ and needle‐based protocols. Transient injection‐site erythema occurred in 30/36 patients (83.3%) and resolved within 48 h. Edema occurred in 28/36 patients (77.8%) and typically resolved within 2–4 days with conservative measures (e.g., cold compress). Ecchymosis occurred in 15/36 patients (41.7%) and resolved within 3–7 days. Mean procedure‐related pain was 3.2 ± 1.8 on a 0–10 VAS.

Delayed nodules were reported in 2/36 patients (5.6%) and resolved with massage by 8 weeks. There were no infections, no vascular compromise events, and no serious adverse events.

### 3.5 Imaging and Representative Cases

Representative clinical photographs (baseline vs. 6 months) are shown in Figures 2, 3, 4, demonstrating improvements consistent with enhanced surface evenness and radiance following treatment. Although not quantitatively analyzed in this study, the imaging illustrates qualitative changes consistent with improved dermal reflectivity/echogenicity after treatment.

## 4 Discussion

Skin quality is increasingly treated as an endpoint distinct from classical volumetric correction or isolated wrinkle reduction. Consensus frameworks emphasize that skin quality includes multiple domains—such as surface evenness, tone evenness, firmness, and glow—that can be influenced by epidermal barrier function, dermal ECM organization, microvascular changes, and subcutaneous support [1, 2]. Because multiple anatomic layers contribute, treatments that act on a single mechanism may produce limited improvements in certain domains. Our findings suggest that a hybrid approach targeting both broad subdermal remodeling (via diluted CaHA) and superficial dermal revitalization (via intradermal PN) can yield meaningful improvements that remain detectable at 6 months.

Importantly, the magnitude of improvement in the photonumeric skin‐quality score (44.4%) indicates more than minimal change and is supported by convergent measures: cheek wrinkle severity improved by 41.7%, and the majority of patients and clinicians selected GAIS categories indicating clear improvement. Notably, no evaluator rated any patient as “Worse,” and patients most frequently selected the higher improvement categories (“Very Much Improved” or “Much Improved”), suggesting that the changes were not merely statistically significant but also clinically recognizable.

CaHA has been used for more than a decade in aesthetic practice, and reviews describe a generally favorable safety profile when appropriate injection depth, technique, and product handling are applied [3, 4]. While CaHA was initially positioned largely as a volumizing filler for deeper planes, its use has evolved toward biostimulatory applications in dilute or hyperdilute form. In these protocols, the aim is not focal volume replacement but rather broad tissue remodeling, with CaHA acting as a scaffold that promotes collagen neosynthesis and improvement in dermal mechanical properties. Consensus guidance supports CaHA use as a biostimulatory agent across the face and body and provides practical recommendations on dilution and plane selection [5].

Clinical studies have explored diluted CaHA for improvement in skin laxity and lines across multiple anatomical regions. For example, a pilot study reported improved neocollagenesis and skin mechanical properties following diluted CaHA injections in the neck and décolletage [6]. A more recent clinical and ultrasonographic assessment demonstrated improvement in clinical scales and increases in dermal thickness after hyperdiluted CaHA treatment in the neck [7]. Although our study did not include objective ultrasound thickness measurements, the direction and magnitude of the skin‐quality improvement at 6 months align with the concept that CaHA can drive durable dermal remodeling.

DOT‐PN (Rejuran, Korea) products are increasingly used in aesthetic medicine as scaffold injectables.

Despite heterogeneity, several higher‐quality clinical studies support DOT‐PN's potential role in wrinkle and skin quality improvement. In a phase III randomized matched‐pairs trial, DOT‐PN filler demonstrated efficacy and durability for correction of crow's feet with an acceptable safety profile compared with hyaluronic acid filler [10]. A systematic review summarizing DOT‐PN studies in aesthetic indications concluded that PN injections show promising outcomes in reducing wrinkles and improving texture and elasticity, with generally mild adverse events, but emphasized the need for additional rigorous trials [9].

The hybrid CaHA–PN protocol evaluated here was designed around complementarity of biological targets and injection planes. Diluted CaHA was placed subdermally using a cannula to allow broad distribution, reduce injection points, and mitigate superficial irregularities. PN was placed intradermally in micro‐aliquots to target the superficial dermal compartment, which is closely linked to perceived radiance and fine surface changes. This plane‐specific strategy may be important in combination approaches: placing CaHA too superficially can increase the risk of visible nodularity or irregularity, whereas placing PN too deeply could reduce its intended biorevitalizing effects.

From a mechanistic viewpoint, CaHA's role as a scaffold and trigger for collagen remodeling can be conceptualized as addressing the structural ‘framework’ of skin quality, while PN may modulate the ‘microenvironment’ that influences hydration, inflammation, and early regenerative signaling. In combination, these pathways may provide both an early perceived improvement in glow and texture and a sustained remodeling effect. Although our study cannot separate the contribution of each component, the sustained improvements at 6 months support the clinical plausibility of synergy, particularly given that single‐agent skin boosters often show diminishing effect over time in real‐world practice.

Combination strategies are increasingly common in aesthetic practice as clinicians attempt to address the multidimensional nature of aging. A related concept is the use of CaHA in combination with hyaluronic acid gels. A recent retrospective report of a premixed CaHA/CMC–hyaluronic acid hybrid filler described improvements in multiple mid‐ and lower‐face aging domains and cheek wrinkle severity at 4 months in a 12‐participant cohort [12]. That study differs from ours in product formulation (premixed hybrid filler vs. separate products) and in intended biological emphasis (hyaluronic acid's viscoelastic and hydrating effects vs. PN's regenerative signaling). Nevertheless, the existence of published outcomes for other CaHA‐based hybrid strategies supports a broader trend toward multimodal injectables for skin quality and suggests that CaHA can be a flexible platform in combination protocols.

Safety is a central consideration when combining injectables, particularly when different planes and delivery tools (cannula and needle) are used in one session. In our cohort, the safety profile was favorable. The most frequent events were transient erythema and edema, which are expected after cannula and needle injections. Ecchymosis occurred in 41.7%, consistent with real‐world bruising rates for procedures that include intradermal micro‐aliquots. Pain scores were low to moderate (mean 3.2/10), supporting tolerability.

Delayed nodules occurred in 2 patients (5.6%) and resolved with massage by 8 weeks. While nodularity is a recognized adverse event for many injectable products, careful plane selection, dilution strategy for CaHA, and gentle low‐pressure delivery may reduce risk. Review data for CaHA note that complications are uncommon when appropriate technique is used but emphasize the importance of anatomical knowledge, avoidance of superficial bolus injection, and readiness to manage adverse events [4]. Importantly, we observed no infections or vascular compromise events. Although absence of vascular events in a cohort of 36 cannot be generalized, it supports the premise that a cannula‐based subdermal approach, combined with careful intradermal micro‐aliquot technique, can be performed safely in experienced hands.

From a patient‐experience standpoint, a single‐session protocol is clinically attractive. Patients often seek treatments that provide visible improvement with minimal downtime and limited clinic visits. In this cohort, high radiance ratings and satisfaction align with this preference. The fixed‐dose approach also improves reproducibility and may be useful for standardizing protocols in future comparative trials.

Representative standardized photographs (Figures 1, 2, 3) provide qualitative support for the measured improvements in skin‐quality scores. In addition, Figure 4 shows a representative cross‐sectional skin imaging screenshot with pseudocolor mapping at baseline and follow‐up. Because the imaging was not quantified and was available only as representative output, it should be interpreted as supportive rather than definitive evidence of structural change. Future studies should integrate standardized quantitative imaging endpoints (e.g., high‐frequency ultrasound measures of dermal thickness or echogenicity) to strengthen mechanistic inference and reduce reliance on photograph‐based scoring.

This study has limitations. First, its retrospective design and lack of a control group prevent causal attribution and do not allow estimation of the incremental benefit of adding PN to CaHA or vice versa. Second, outcomes were primarily photograph‐based. Although the primary endpoint used a validated photonumeric skin‐quality scale and ratings were performed by blinded dermatologists [14, 18], objective instrumental endpoints (e.g., elasticity, hydration, 3D topography, or quantitative ultrasound) were not systematically collected. Third, this was a single‐center study with all injections performed by one injector; while this improves internal consistency, it may limit generalizability to other techniques or practice settings. Fourth, the cohort was predominantly female, which is common in cosmetic practice but may limit inferences to male patients. Finally, longer‐term durability beyond 6 months was not assessed. In addition, the use of the Modified Fitzpatrick Wrinkle Scale, which is validated for nasolabial folds, was adapted for the assessment of cheek wrinkles in this study. Although this approach enabled consistent within‐subject comparisons, it may limit direct comparability with studies using region‐specific validated scales.

Prospective trials are needed to validate and extend these findings. Randomized controlled designs comparing CaHA–PN hybrid treatment with CaHA‐only and PN‐only regimens would clarify the incremental contribution of each component. Split‐face randomized designs could be particularly informative because they control for individual‐level confounders in skin type, lifestyle, and baseline aging. Future work should also standardize injection mapping and include multimodal endpoints: validated photonumeric scales, objective measures of skin mechanics (cutometry), barrier function (transepidermal water loss), hydration (corneometry), and imaging‐based structural measures (high‐frequency ultrasound or other cross‐sectional techniques). Finally, comparative dosing schedules (single vs. repeated sessions) and alternative dilution ratios may identify regimens that maximize efficacy while maintaining safety.

In summary, our real‐world findings support the hypothesis that the plane‐specific combination of diluted CaHA and intradermal PN can provide meaningful improvements in multiple skin‐quality domains with acceptable tolerability. These data add to the growing literature on combination injectable strategies for skin quality and provide a foundation for prospective comparative research.

## 5 Conclusion

In a consecutive cohort of 36 patients, a single‐session hybrid skin booster protocol combining subdermal diluted CaHA and intradermal PN was associated with significant improvements in facial skin quality and cheek wrinkle severity at 6‐month follow‐up. Clinician and patient GAIS ratings indicated improvement in most patients, and overall satisfaction was high. Adverse events were predominantly mild and transient injection‐site reactions, with no serious complications observed. These findings support the clinical feasibility of plane‐specific CaHA–PN hybrid skin boosting and highlight the need for prospective controlled trials to define comparative efficacy and optimize treatment parameters.

## Author Contributions

Writing‐original draft preparation: Andrea Lazzarotto, Stefania Guida, Jonathan Kadouch. Writing‐review and editing: Andrea Lazzarotto, Nabil Fakih‐Gomez, Nicola Zerbinati. Visualization: Luigi Colombo, Massimo Vitale, Mariana César Corrêa. Supervision: Kyu‐Ho Yi.

## Funding

The authors have nothing to report.

## Ethics Statement

All treatments were performed in adherence with the Declaration of Helsinki and in accordance with local regulations and standards of good clinical practice. This article does not contain any studies with animals performed by any of the authors.

## Consent

All patients included in this study provided written informed consent for access to their medical records and for the use of their data (and clinical images, where applicable) for research and publication purposes.

## Conflicts of Interest

Andrea Lazzarotto, Stefania Guida, Nabil Fakih‐Gomez and Jonathan Kadouch are consultants for Merz Aesthetics (Frankfurt, Germany). Andrea Lazzarotto is also a consultant for Fidia Pharma. The other authors declare no conflicts of interest.

## Data Availability

Data sharing not applicable to this article as no datasets were generated or analysed during the current study.
